# Physical activity and depressive symptoms in young adults: a moderated mediation model of neighborhood trust and growth mindset

**DOI:** 10.3389/fpubh.2026.1772982

**Published:** 2026-06-10

**Authors:** Hui Wang, Zhiyi Li

**Affiliations:** 1 School of Humanities and Social Science, Xi’an Jiaotong University, Xi’an, China; 2 School of Public Administration, Xi'an University of Architecture and Technology, Xi’an, China

**Keywords:** depressive symptoms, growth mindset, neighborhood trust, physical activity, young adults

## Abstract

**Introduction:**

While physical activity is widely recognized as an effective intervention for mitigating depressive symptoms among young adults, the psychosocial mechanisms mediating these effects are not yet fully elucidated. Grounded in China’s national fitness campaign and the development of 15-minute community living circles, this study proposes a socio-cognitive integration model to examine community-level mechanisms linking physical activity to reduced depressive symptoms.

**Methods:**

Drawing on data from the 2022 China Family Panel Studies (CFPS), this study employed generalized structural equation modeling (GSEM) and multiple moderation analyses to examine the mediating role of neighborhood trust and the moderating effect of growth mindset in young adults aged 18–44.

**Results:**

The mean score for depressive symptoms was 5.67, while the average frequency of physical activity was 1.67. Physical activity directly improved mental health and indirectly reduced depressive symptoms through enhanced neighborhood trust. Notably, growth mindset positively moderated this mediation, with stronger effects observed among individuals endorsing higher growth mindset levels.

**Conclusion:**

These findings advance psychosocial explanations for physical activity benefits and highlight the value of community environment optimization and mindset-customized interventions.

## 1 Introduction

Depressive symptoms result from complex interactions between individual psychological states and societal structural pressures. Common manifestations include exhaustion, depressed mood, lack of concentration, loss of appetite, disinterest in daily activities, sleep disturbances, and feelings of worthlessness and guilt (1, 2). Although these symptoms do not always indicate clinical depression, they can still significantly impair daily functioning and reduce quality of life (3, 4).

Recent studies underscore the heightened mental health vulnerability of young adults, who face unique developmental challenges. From a role transition perspective, this period involves shifting from education to employment, introducing new responsibilities and societal expectations. Adapting to unfamiliar environments during these transitions may profoundly impact psychological well-being (5). Concurrently, this life stage demands increased personal autonomy as young adults make independent career and relationship decisions. While autonomy can be empowering, it may also generate substantial stress (6). Furthermore, ongoing identity exploration and self-concept formation make this group especially sensitive to environmental and interpersonal fluctuations (7, 8).

These cumulative factors elevate the risk of depressive symptoms among young adults. According to the Report on National Mental Health Development in China (2021–2022), 14.8% of surveyed young adults exhibited depressive symptoms, with 10.8% at risk for mild depression and 4.0% meeting thresholds for severe depression (9). Notably, this prevalence is substantially higher than that of other age groups. A recent meta-analysis further indicates a 4% increase in depression prevalence among Chinese young adults in the post-pandemic era (10). The resulting negative effects have extended beyond mental health issues to multiple aspects, including academic performance, career development, and social relationships (11, 12). Given these findings, developing cost-effective and accessible interventions tailored to young adults has become a critical priority for both public health research and policy.

Among various approaches, physical activity has received growing attention due to its health benefits, practical advantages and research-practice gap. Physical activity demonstrates multidimensional health benefits, with meta-analyses confirming its significant alleviation of depressive symptoms and improvement of overall well-being (13, 14). In addition to its health benefits, physical activity also offers practical advantages for young adults, combining low accessibility barriers with remarkable adaptability to diverse lifestyles (15). However, a concerning gap persists between actual physical activity participation and recommended levels (16, 17). Therefore, to bridge this gap and better serve the development of public health interventions for young adults, in-depth research on the health mechanisms of physical activity is of great theoretical and practical value.

Research has found that physical activity can mitigate young adults’ depressive symptoms (18, 19), through its positive roles on emotional regulation, sleep quality, self-efficacy, and social connectedness (20–23). However, existing literature primarily examines physical activity through a micro-level lens, focusing on its direct antidepressant effects at the individual level. Few studies have investigated its meso-level psychosocial mechanisms, particularly how it enhances neighborhood trust. Within the framework of community social capital, regular physical activity strengthens neighborhood ties by shared experiences, thereby increasing mutual trust among residents (24). In a high-trust community, individuals experience greater belongingness and reciprocal help-seeking behaviors, ultimately improving mental health (25–27). Furthermore, these benefits may vary substantially depending on individual mindset differences (28), a factor often neglected in intervention designs.

This study aims to investigate how neighborhood trust and growth mindset synergistically enhance physical activity’s mental health benefits. The article proceeds as follows: First, we elucidate the theoretical and practical significance of these psychosocial moderators. Next, we formulate testable hypotheses by integrating social capital theory with cognitive-behavioral frameworks, followed by a detailed description of our mixed-methods design. After presenting stratified analyses that demonstrate both mediating and moderating effects, we discuss implications for developing culturally contextualized interventions, concluding with methodological considerations and future directions for implementation science.

## 2 Research hypotheses

### 2.1 Physical activity and depressive symptoms

Physical activity is regarded as a primary intervention for alleviating depressive symptoms in young adults. First and foremost, it improves brain function (29, 30), enhancing stress coping abilities and mental clarity (31, 32). Additionally, physical activity provides immediate emotional relief by shifting focus away from negative thoughts, while also promoting long-term optimism (33–35). Moreover, group activities mitigate isolation and build self-worth through expanded support networks (36–38). A randomized controlled trial further reveals that 8 weeks of structured physical activity leads to significant improvements in mental health among young adults at risk for depression (39). Based on this evidence, we propose Hypothesis 1:

*H1*: Physical activity is negatively associated with depressive symptoms among young adults.

### 2.2 Physical activity, neighborhood trust and depressive symptoms

Existing studies have established a robust foundation for understanding the health benefits of physical activity. However, the mechanisms through which neighborhood trust mediates its effects on mental health remain underexplored. Influenced by transportation convenience, time costs, and sports facility availability (40, 41), most young adults engage in physical activity within their local communities. From a social capital perspective, this communal participation pattern creates unique opportunities for trust-building through repeated social interactions and shared experiences (42). This underscores the importance of studying how physical activity fosters trust at the community level, where collective efficacy and network closure may amplify its psychosocial benefits (43). Trust, a psychological state of accepting vulnerability based on positive expectations of another’s intentions, operates as a complex socio-psychological mechanism governed by reciprocity and shaped by social contexts (44). Within communities, this manifests as neighborhood trust and refers to residents’ confidence in the reliability, honesty, and goodwill of neighbors (42). According to social capital theory, regular face-to-face interactions can improve trust by increasing reciprocity norms and shared identity (45, 46). Consequently, the community-based nature of physical activity fosters neighborhood interactions, thus mitigating social isolation (24, 47), ultimately reinforcing neighborhood trust and relational stability (46, 48). Previous empirical studies have supported this theoretical perspective. Research among Canadian youth groups has demonstrated that physical activity can effectively promote community engagement and trust (49). A study by Lin et al. further revealed that increased community sports participation leads to significant improvements in neighborhood trust levels (50).

Based on Berkman’s Social Integration-Health Model, community social capital significantly improves individuals’ health behaviors and psychological well-being through social support and psychological resources (51). The model highlights the protective role of neighborhood trust in mitigating depressive symptoms, which has been widely supported by cross-cultural studies. For instance, research on Indonesian residents showed a significant negative correlation between neighborhood trust and depressive symptoms (52). Similarly, Bassett et al.’s Canadian study found that individuals with high neighborhood trust had a 17% lower risk of depressive symptoms compared to their low-trust counterparts (53). The growing complexity of social risks has reinforced the importance of neighborhood trust. During the COVID-19 pandemic, neighborhood trust proved indispensable for maintaining daily functioning and mental health (54, 55). Beyond crisis contexts, high-trust neighborhood environments buffer against work and life stressors, thereby enhancing psychological well-being (56). Empirical studies further demonstrate that neighborhood trust improves mental health by reducing daily conflicts (57–59). Thus, we hypothesize (H2):

*H2*: Physical activity enhances neighborhood trust, thereby mitigating depressive symptoms among young adults.

### 2.3 Physical activity, growth mindset and neighborhood trust

To understand how physical activity affects depressive symptoms through neighborhood trust, a key question arises: Which individuals derive greater benefits from this pathway? We propose growth mindset as a moderating factor to explain these individual differences. The growth mindset, a psychological construct pioneered by Dweck, refers to an individual’s belief that personal abilities (e.g., cognitive skills, social competence) can be developed through effort, learning, and experience (60). Its defining characteristics include plasticity of abilities, challenge-embracing and effort orientation (61, 62). Research indicates that a growth mindset not only influences social behaviors but also molds the way individuals build their social capital. For instance, Zander et al. found that students endorsing a growth mindset were more inclined toward cooperative learning and establishing positive peer relationships, demonstrating stronger social adaptability (63). Complementing these findings, studies on physical activity revealed that individuals with a growth mindset showed increased cooperative tendencies and communication during sports activities, exhibiting elevated social engagement (64).

Building on existing evidence that growth mindset individuals have proactive social tendencies, we posit that the difference in mindsets alters neighborhood trust accumulation pathways in community-based physical activity settings. On one hand, growth mindset individuals are more likely to proactively greet neighbors, exchange fitness tips, and share experiences during exercise, thereby enhancing mutual understanding among community members (65–67). On the other hand, when conflicts or disagreements arise in group activities, they tend to adopt a constructive approach, resolving issues through self-reflection rather than avoidance (68). Such frequent and high-quality interactions can foster familiarity and emotional closeness, ultimately strengthening neighborhood trust (69). Therefore, we propose Hypothesis 3:

*H3*: The positive association between physical activity and neighborhood trust is moderated by growth mindset, such that the effect is significantly enhanced for individuals with a strong growth mindset.

### 2.4 Theoretical framework

**Figure 1:**
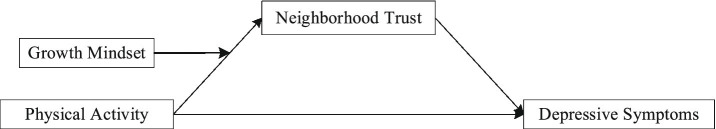
Theoretical framework of the socio-cognitive integrated model for physical activity and depressive symptoms.

Building on existing theoretical foundations, this study develops a socio-cognitive integration model (Figure 1) that systematically elucidates the psychosocial pathways through which physical activity influences depressive symptoms in young adults. The model proposes two distinct yet complementary mechanisms: first, physical activity exerts both direct effects on depressive symptom reduction and indirect effects through enhanced neighborhood trust; second, growth mindset significantly moderates the physical activity-neighborhood trust relationship, with the trust-building effect being substantially stronger among individuals exhibiting more pronounced growth mindset characteristics. This integrative framework advances current understanding by specifying the dynamic interplay between behavioral, social, and cognitive factors in mental health promotion.

## 3 Methods

### 3.1 Sample

The data for this study were sourced from the 2022 wave of the China Family Panel Studies (CFPS), a nationally representative longitudinal survey administered by Peking University. The CFPS employed a multistage stratified sampling method to select households and individuals from 140 counties across 25 provinces in China. First, county-level administrative units were selected as primary sampling units through probability proportional to size (PPS) systematic sampling, using national administrative division data. In the second-stage sampling, villages or neighborhood committees were extracted as secondary sampling units from the selected county-level units. During the final sampling stage, a systematic random sampling method was employed to select households and individuals from grassroots communities.

As a comprehensive social tracking project, the CFPS monitors multidimensional changes across individual, household, and societal levels, with six completed waves since its inception. The survey’s scientific validity and methodological rigor have been well established in previous studies (70, 71). Our selection of the 2022 cross-sectional data was primarily motivated by its first-time incorporation of the growth mindset assessment items, which were essential for testing the psychosocial moderation hypotheses. The 2022 wave of CFPS included 26,435 individuals (51% male and 49% female), with 59% aged 15–59, 25% aged 60 or older, and fewer than 10% having higher education. This distribution aligns closely with China’s national census data, ensuring representativeness. After excluding participants outside the target age range and those with missing data, the final analytical sample comprised 8,834 individuals.

### 3.2 Measurements

#### 3.2.1 Dependent variable: depressive symptoms

Depressive symptoms were assessed using the 8-item Center for Epidemiologic Studies Depression Scale (CES-D8), a psychometrically validated instrument widely employed in cross-cultural mental health research targeting youth populations (72, 73). The scale measures the frequency of eight distinct affective and behavioral manifestations over the past week: (1) depressed mood, (2) psychomotor retardation, (3) sleep disturbances, (4) positive affect (reverse-scored), (5) feelings of loneliness, (6) life satisfaction (reverse-scored), (7) sadness, and (8) hopelessness. A sample item from the CES-D is: “I felt depressed during the past week.” Responses were captured on a 4-point Likert-type scale ranging from 1 (“rarely or none of the time “) to 4 (“most or all of the time “). In accordance with standard scoring protocols, positive items were reverse-coded, and all item scores were subsequently subtracted by 1 to yield a 0–3 metric. The transformed scores were then aggregated to generate a composite depressive symptoms index with a potential range of 0–24 (Cronbach’s *α* = 0.95), where elevated scores correspond to greater depressive symptoms.

#### 3.2.2 Independent variable: physical activity

Physical activity was assessed using a self-reported measure of leisure-time exercise frequency, adapted from the International Physical Activity Questionnaire (IPAQ) (74, 75), which has demonstrated reliability and validity. Participants responded to the item: “How often did you engage in sports or exercise during the past 12 months?” on a 7-point Likert-type scale ranging from 1 (“less than once per month”) to 7 (“twice daily or more”), with higher scores reflecting greater activity frequency. The original “never participated” response (coded as 8) was recoded as 0 to maintain ordinal consistency and enhance analytical interpretability.

#### 3.2.3 Mediator: neighborhood trust

Neighborhood trust was assessed using the single-item question: “How much do you trust your neighbors?” This measurement approach has been extensively validated across diverse cultural settings (76, 77). Respondents rated their level of trust on an 11-point Likert-type scale ranging from 0 (“complete distrust”) to 10 (“complete trust”).

#### 3.2.4 Moderator: growth mindset

Growth mindset was assessed using a single-item measure adapted from the Implicit Theories of Intelligence Scale (78, 79). The item stated: “Regardless of current intelligence level, people can always change their intelligence.” Respondents rated their agreement (1 = “strongly disagree” to 5 = “strongly agree”) with a statement concerning the malleability of intellectual ability. Existing literature supports the use of single-item measurement approach when targeting precise, narrowly-focused constructs (80, 81).

#### 3.2.5 Control variables

Consistent with previous mental health research (82, 83), we controlled for several covariates: age (continuous in years), gender (1 = male; 0 = female), marital status (1 = stable relationship; 0 = otherwise), education level (1 = “no formal education” to 8 = “doctoral degree”), residence type (1 = urban; 0 = rural), political affiliation (1 = China Communist Party member; 0 = non-member), relative income (5-point scale with higher values indicating greater relative income), presence of chronic disease (1 = yes; 0 = no) and religious affiliation (1 = religious; 0 = non-religious).

### 3.3 Analytical strategy

This study employed a comprehensive analytical framework to examine the hypothesized relationships. The analysis proceeded through four systematic stages: First, descriptive statistics were computed to characterize the distributional properties of all study variables. Second, multiple linear regression analysis was conducted to assess the direct effects of physical activity and neighborhood trust on depressive symptoms. Third, generalized structural equation modeling was implemented to investigate the potential mediating role of neighborhood trust in the association between physical activity and depressive symptoms. Finally, to rigorously test the moderating effect of growth mindset, we employed multiple complementary approaches including interaction term analysis, the seemingly unrelated estimation (Suest) test, and Chow test for parameter stability. All analyses were conducted using Stata 16.0.

## 4 Results

### 4.1 Descriptive analysis

The study sample (*N* = 8,834) comprised young adults with a mean age of 31.0 years and balanced gender distribution (50.2% male). Geographically, more than half of the participants resided in urban areas. Less than 11% were Communist Party members. The relative income level was predominantly moderate, consistent with the young adult demographic. Given the study’s focus on youth, the prevalence of chronic diseases was low (6.5%).

Regarding core study variables, the mean score for depressive symptoms was 5.668, while the average frequency of physical activity was 1.671. Neighborhood trust exhibited a relatively high mean score (6.357), and growth mindset averaged 3.398 (Table 1).

**Table 1:** Basic characteristics of related variables.

Variable	Mean	Std	Range
Depressive symptoms	5.668	3.805	0 ~ 24
Physical activity	1. 671	2.053	0 ~ 7
Neighborhood trust	6.375	2.058	0 ~ 10
Growth mindset	3.398	1.133	1 ~ 5
Age	31.602	7.199	18 ~ 44
Gender	0.502	0.500	0,1
Education	3.909	1.402	1 ~ 8
Residence	0.562	0.562	0,1
Marriage	0.619	0.485	0,1
Political affiliation	0.107	0.309	0,1
Religious affiliation	0.014	0.121	0,1
Relative income level	2.808	0.936	1 ~ 5
Chronic disease status	0.065	0.247	0,1

### 4.2 Mediation effect analysis

Table 2 reports four regression models for depressive symptoms. Model 1 demonstrated the effects of control variables on depressive symptoms among young adults. Consistent with prior research (84–86), lower depressive symptoms were observed in individuals who were younger, male, highly educated, in stable relationships, with higher incomes, and without chronic diseases.

**Table 2:** Multiple linear regression models predicting depressive symptoms.

Variable	Model 1	Model 2	Model 3	Model 4
Age	0.022^**^	0.022^**^	0.017^***^	0.027^***^
	(0.008)	(0.008)	(0.004)	(0.008)
Gender	−0.424^***^	−0.376^***^	0.266^***^	−0.295^***^
	(0.081)	(0.081)	(0.044)	(0.080)
Education	−0.324^***^	−0.266^***^	0.050^**^	−0.251^***^
	(0.032)	(0.033)	(0.018)	(0.032)
Residence	−0.078	−0.055	−0.263^***^	−0.136
	(0.085)	(0.085)	(0.046)	(0.084)
Marriage	−0.284^**^	−0.315^**^	0.255^***^	−0.237^*^
	(0.108)	(0.108)	(0.059)	(0.107)
Political affiliation	−0.147	−0.094	0.150^*^	−0.046
	(0.131)	(0.131)	(0.071)	(0.129)
Religious affiliation	0.494	0.515	−0.387^*^	0.393
	(0.323)	(0.322)	(0.176)	(0.317)
Relative income level	−0.589^***^	−0.583^***^	0.271^***^	−0.497^***^
	(0.043)	(0.042)	(0.023)	(0.042)
Chronic disease status	1.145^***^	1.194^***^	−0.286^***^	1.103^***^
	(0.157)	(0.157)	(0.086)	(0.155)
Physical activity		−0.176^***^	0.048^***^	−0.161^***^
		(0.021)	(0.011)	(0.020)
Neighborhood trust				−0.315^***^
				(0.019)
_cons	8.388^***^	8.394^***^	4.660^***^	9.874^***^
	(0.291)	(0.290)	(0.159)	(0.300)
*N*	8,834	8,834	8,834	8,834
adj. *R*^2^	0.047	0.055	0.036	0.083

Standard errors in parentheses. ^*^p < 0.05, ^**^p < 0.01, ^***^p < 0.001.

Model 2, building upon Model 1 by incorporating physical activity, revealed a significant negative association between regular physical activity and depressive symptoms (*β* = −0.176, *p* < 0.001). Young adults with higher physical activity participation demonstrated lower levels of depressive symptoms relative to their fewer active counterparts. Model 3 further indicated that physical activity positively predicted neighborhood trust (*β* = 0.048, *p* < 0.001). Young adults who engaged in regular physical activity had higher levels of trust toward their neighbors compared to others (Table 2).

**Figure 2:**
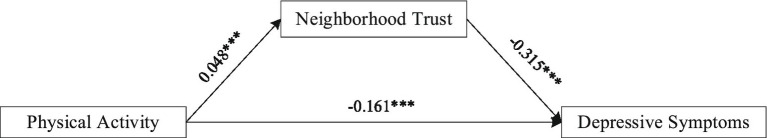
The mediating role of neighborhood trust in the influence of physical activity on depressive symptoms. ^***^ *p* < 0.001.

Model 4 results demonstrated that neighborhood trust significantly reduced depressive symptoms (*β* = −0.315, *p* < 0.001). Supportive community surroundings served as a protective habitat that mitigates anxiety and depressive symptoms among young residents. Furthermore, the attenuation of physical activity’s coefficient from −0.176 in Model 2 to −0.161 in Model 4 indicated that the influence of physical activity on depressive symptoms was partial mediated through neighborhood trust. We formally tested this mediation effect using generalized structural equation modeling (GSEM), with the complete pathway analysis presented in Figure 2.

Figure 2 clearly illustrated the social mechanism through which physical activity influenced depressive symptoms at the community level. The path analysis revealed a significant positive association between physical activity frequency and neighborhood trust (*β* = 0.048, *p* < 0.001). This elevated level of neighborhood trust, in turn, demonstrated a substantial negative relationship with depressive symptoms (*β* = −0.315, *p* < 0.001), suggesting a sequential mediation process.

### 4.3 Moderating effect test

To rigorously examine the moderating effect of growth mindset on the relationship between physical activity and neighborhood trust, we conducted comprehensive analyses using interaction terms, the *Suest* test, and the *Chow* test (Table 3). The results demonstrated consistent and robust evidence for this moderating effect: the interaction term was statistically significant (*p* < 0.05), the *Suest* test rejected the null hypothesis (*χ*^2^ = 3.22, *p* = 0.073), and the Chow test showed significant structural differences between groups (*F* = 30.91, *p* = 0.000). These findings indicated that individuals with stronger growth mindset are particularly adept at leveraging the social benefits of physical activity. They are more likely to initiate social interactions during physical activity and subsequently transform these encounters into valuable weak-tie networks within their neighborhoods. This suggests that growth mindset plays a crucial role in facilitating the translation of physical activity into broader social capital development at the community level.

**Table 3:** Statistical test of moderating effects.

Test type	Interaction analysis	*Suest* test	Chow test
Statistics	2.02	3.22	30.91
*p* value	0.043	0.073	0.000

## 5 Discussion

### 5.1 Effects of physical activity on depressive symptoms in young adults

Physical activity was negatively correlated with depressive symptoms, supporting hypothesis 1. This finding aligns with previous studies (18, 87). Physical activity not only mitigates physical health issues (e.g., obesity and hypertension) among young adults but also alleviates mental stress and depression. While the mental health disparity between physically active and inactive young adults appears modest in the initial stages, the stratification of physical activity participation intensity may mirror the distribution patterns of other health-related social resources (88–90). Young adults with regular physical activity participation can take advantage of the opportunities presented by the era, such as the popularization of community health facilities and the development of sports social platforms. Therefore, they may maintain their advantages in mental health throughout their life course. This cumulative advantage can play a lasting protective role in coping with workplace stress, social anxiety, and building psychological resilience (91–93).

Neighborhood trust mediated the relationship between physical activity and reduced depressive symptoms, confirming hypothesis 2. This finding aligns with social capital research (94, 95). While prior studies have primarily focused on how physical activity strengthens peer-based social networks and support, less attention has been paid to its effects at the community level. Our study demonstrated that the social capital effects of physical activity extend even to unfamiliar neighbors, and this enhanced neighborhood trust significantly improves young adults’ mental health. Existing research on the health effects of social capital has predominantly highlighted the protective role of strong ties in collectivist cultural contexts (96, 97). However, our findings underscore the growing importance of weak ties in promoting the well-being of the Chinese younger generation (98). This paradigm shift reflects two fundamental aspects of rapid social transition. Firstly, the progressive weakening of traditional kinship and geographical bonds has elevated community-based weak ties as alternative support mechanisms. Secondly, unlike previous generations who primarily faced material deprivation, contemporary young adults experience mental health challenges predominantly stemming from social comparison and achievement competition (99, 100). Weak ties, characterized by minimal emotional demands and limited social expectations, emerge as an effective buffer against these modern psychological pressures (101, 102). The positive role of reciprocal trust among neighbors is evident in young populations, suggesting that community-level support policies should not be limited to the “older adults and children” often emphasized in current Chinese policy discourse.

This study further elucidates the protective mechanisms of physical activity and neighborhood trust for youth mental health from the perspective of “the nearby.” The theory posits that contemporary society is experiencing the disappearance of the nearby, a phenomenon where traditional community bonds erode under digitalization and urbanization (103). Consequently, this dramatic shift in social ecology has plunged young people into a dilemma of emotional isolation (104). Community-based physical activity emerges as a crucial medium for nearby reconstruction. Through regular interactions in communities, young people can reweave localized social networks, thus nurturing neighborhood trust. The resulting physical activity–trust–health pathway effectively rebuilds socioecological support systems, enhancing agency and alleviating depressive symptoms.

The third hypothesis that growth mindset significantly moderates the effect of physical activity on neighborhood trust was supported. Results revealed a stronger positive association between physical activity and neighborhood trust among growth-minded individuals. This breakthrough extends growth mindset research from its traditional educational domain to social capital and public health outcomes (105, 106), demonstrating its role as a community behavioral catalyst. The moderating effect can be interpreted from cognitive patterns and attribution differences (107). Although neighbors occupy the periphery in the differential order pattern (108), growth-minded individuals actively transform weak ties into meaningful connections during physical activities. Additionally, this mindset influences how individuals perceive the indifference among neighbors, which is a typical issue in contemporary urban communities (109–111). Growth-minded individuals tend to attribute such indifference to situational barriers (e.g., limited interaction opportunities) rather than to the inherent unfriendliness of neighbors. Crucially, this cognitive-behavioral divergence may exacerbate community participation inequalities. Growth-minded individuals benefit from a self-reinforcing cycle where initial social successes enhance subsequent engagement (112, 113), while fixed-mindset peers risk network deprivation. These findings position growth mindset as a social lubricant in the community, thus converting physical activity into relational infrastructure for trust cultivation.

### 5.2 Implications and future research

Our findings provide actionable insights for improving youth mental health through community-level interventions. Urban planners should prioritize green space integration and walkable infrastructure to stimulate physical activity and spontaneous social encounters, thereby enhancing both exercise adherence and neighborhood trust. We advocate for community fitness initiatives that simultaneously improve physiological health and reinforce social bonds. Crucially, these interventions must employ tailored strategies to accommodate individual variations in personality traits and cognitive preferences, guaranteeing equitable participation across diverse youth demographics.

Several limitations should be acknowledged. The community-level analysis focused solely on neighborhood trust without incorporating other potential influencing factors (e.g., community participation and sense of belonging). Additionally, measurement limitations require attention. For example, growth mindset was assessed using a single-item measure, potentially failing to capture the full complexity of this construct. Moreover, the cross-sectional design precludes causal inferences between physical activity and depressive symptoms. Future research could advance in three key directions: First, the comprehensive community models that incorporate multidimensional indicators would provide more robust insights. Second, measurement validity could be strengthened through multi-item scales. Third, implementing longitudinal designs would help elucidate the dynamic relationships among physical activity, neighborhood trust, and depressive symptoms.

## Data Availability

Publicly available datasets were analyzed in this study. This data can be found at: https://www.isss.pku.edu.cn/cfps/.
